# Cultivated Human Vaginal Microbiome Communities Impact Zika and Herpes Simplex Virus Replication in *ex vivo* Vaginal Mucosal Cultures

**DOI:** 10.3389/fmicb.2018.03340

**Published:** 2019-01-14

**Authors:** Megan H. Amerson-Brown, Aaron L. Miller, Carrie A. Maxwell, Mellodee M. White, Kathleen L. Vincent, Nigel Bourne, Richard B. Pyles

**Affiliations:** ^1^Graduate School of Biomedical Sciences, The University of Texas Medical Branch at Galveston, Galveston, TX, United States; ^2^Department of Pediatrics, The University of Texas Medical Branch at Galveston, Galveston, TX, United States; ^3^Department of Microbiology and Immunology, The University of Texas Medical Branch at Galveston, Galveston, TX, United States; ^4^Department of Obstetrics and Gynecology, The University of Texas Medical Branch at Galveston, Galveston, TX, United States

**Keywords:** vaginal microbiome, Zika virus, herpes simplex virus type 2, women’s health, vaginal mucosa

## Abstract

The human vaginal microbiome (VMB) is a complex bacterial community that interacts closely with vaginal epithelial cells (VECs) impacting the mucosal phenotype and its responses to pathogenic insults. The VMB and VEC relationship includes nutrient exchange and regulation of signaling molecules that controls numerous host functions and defends against invading pathogens. To better understand infection and replication of sexually transmitted viral pathogens in the human vaginal mucosa we used our *ex vivo* VEC multilayer culture system. We tested the hypothesis that selected VMB communities could be identified that alter the replication of sexually transmitted viruses consistent with reported clinical associations. Sterile VEC multilayer cultures or those colonized with VMB dominated by specific *Lactobacillus* spp., or VMB lacking lactobacilli, were infected with Zika virus, (ZIKV) a single stranded RNA virus, or Herpes Simplex Virus type 2 (HSV-2), a double stranded DNA virus. The virus was added to the apical surface of the cultured VEC multilayer to model transmission during vaginal intercourse. Viral replication was measured 48 h later by qPCR. The results indicated that VEC cultures colonized by VMB containing *Staphylococcus* spp., previously reported as inflammatory, significantly reduced the quantity of viral genomes produced by ZIKV. HSV-2 titers were decreased by nearly every VMB tested relative to the sterile control, although *Lactobacillus* spp.-dominated VMBs caused the greatest reduction in HSV-2 titer consistent with clinical observations. To explore the mechanism for reduced ZIKV titers, we investigated inflammation created by ZIKV infection, VMB colonization or pre-exposure to selected TLR agonists. Finally, expression levels of human beta defensins 1–3 were quantified in cultures infected by ZIKV and those colonized by VMBs that impacted ZIKV titers. Human beta defensins 1–3 produced by the VEC showed no association with ZIKV titers. The data presented expands the utility of this *ex vivo* model system providing controlled and reproducible methods to study the VMB impact on STIs and indicated an association between viral replication and specific bacterial species within the VMB.

## Introduction

The vaginal mucosa supports an intricate ecosystem regulated by interactions between microbial communities and vaginal epithelial cells (VECs). Microbial communities associated with an optimal vaginal mucosa establish a symbiotic relationship with VEC to exchange nutrients and create an environment that protects against invading pathogens (Ma et al., 2012; Pyles et al., 2014). A vaginal microbiome (VMB) that contains an abundance of *Lactobacillus* spp. is associated with health (Ravel et al., 2011). In contrast, microbiomes that are deficient in *Lactobacillus* spp. and have a predominance of Gram negative organisms are associated with bacterial vaginosis (BV) and inflammation of the vaginal mucosa (Ma et al., 2012). Such microbial communities, including those associated with symptomatic BV, are correlated clinically and in model systems with increased viral replication for sexually transmitted viruses including HIV and HSV-2 (Boris and Barbés, 2000; Brotman, 2011; Pyles et al., 2014).

Clinical studies of the VMB have shown that the relationships between bacteria and VEC impact the physical and chemical barriers that serve as a primary defense mechanism against invading pathogens (Boris and Barbés, 2000; Brotman, 2011; Pyles et al., 2014; Martin and Marrazzo, 2016). VMB communities can, among other functions, alter innate immune responses, contribute antimicrobial compounds and directly alter the local environment by changing the cells’ susceptibility to pathogens (Boris and Barbés, 2000; Anahtar et al., 2015). The VMB profile, as well as individual bacteria in the vaginal community, have been shown to impact acquisition and in some cases infection outcomes of STI viruses (Boris and Barbés, 2000; Brotman, 2011; Pyles et al., 2014; Gosmann et al., 2017). To date, these studies have not included analyses of sexually transmitted Zika virus (ZIKV).

The recent outbreak in South and Central America established that ZIKV can be transmitted through sexual contact (Arsuaga et al., 2016; Brooks et al., 2016) raising concerns regarding epidemiological control (Coelho et al., 2016). Until recently, ZIKV was considered a neglected tropical disease and little research had been done on the potential for sexual transmission (Coelho et al., 2016). ZIKV is transmitted commonly through the bite of an infected *Aedes aegypti* mosquito (the primary vector) but infections have been diagnosed in humans living in areas where this mosquito species is not found (Foy et al., 2011; Faye et al., 2013; Fréour et al., 2016; Hills et al., 2016). In infected men, persistent and high titers of ZIKV are found in seminal fluid samples (Mansuy et al., 2016). Ongoing efforts to better understand human infection have established that ZIKV can be transmitted to women following sexual intercourse with an infected man leading to significant sequelae including neonatal impacts to the neurological system and microcephaly (de Paula Freitas et al., 2016).

Females infected through mosquitoes or by sexual transmission have detectable ZIKV in vaginal secretions (Murray et al., 2017) suggesting that ZIKV likely infects VEC. Consistent with this theory, ZIKV has been reported to productively infect many different cell types (El Costa et al., 2016; Hirsch et al., 2017), including epithelial cells (Hamel et al., 2015). Direct evidence of vaginal epithelial infection has been reported in mice (Tang et al., 2016), rhesus macaques (Carroll et al., 2017) and a single study in human VECs cultured in monolayer format (Fink et al., 2018), supports localized vaginal mucosal infection in humans but direct evidence is lacking. Likewise, there have been no studies reported to associate or evaluate the impact of VMB communities on ZIKV infection. Understanding the replication of ZIKV in VEC in the presence of established VMB communities would support the identification of bacterial species or microbial products that could decrease the viral burden in the vaginal mucosa and may identify molecular pathways involved in inhibiting replication of the virus.

To begin studies addressing the impact of selected VMB communities on ZIKV infection, we employed our previously described polarized VEC multilayer culture model that creates the necessary environment for colonization with VMB communities transplanted from donors without vaginal infections (Rose et al., 2012; Pyles et al., 2014). In this VEC culture system, an air-interfaced apical surface is created after cells form monolayers in a transwell insert. The air interface stimulates polarization and multilayer formation creating a better model of the vaginal mucosa. The chamber system also enhances study of viral replication and subsequent release of the virus into the basal chamber, modeling viral dissemination away from the mucosa. Using this human vaginal model colonized by VMB communities, we compared the impact that a distinct VMB community has on the susceptibility to infection by ZIKV, a single stranded RNA virus, and HSV-2, a double stranded DNA virus. Testing of 35 distinct VMB communities collected from 22 healthy women identified communities and organisms that impacted these modeled sexually transmitted infections. Collectively, these studies confirmed that after infection of ZIKV, VEC supported viral replication before being systemically released into the basal chamber. VMB colonization established that distinct communities altered viral replication of ZIKV and HSV-2. In addition, ZIKV infection did not cause significant changes in established VMBs. Mechanistically, cytokine levels, activation of specific toll-like receptors (TLRs) and human beta defensin production were not found to be solely responsible for altered ZIKV outcomes. These finding support future studies to understand the pathways involved in enhancing or suppressing ZIKV replication related to the identified VMB profiles.

## Materials and Methods

### Ethics Statement

The collection of VMB samples from volunteers was approved by the University of Texas Medical Branch’s institutional review board. Adult subjects provided written informed consent prior to the collection of vaginal swabs. Each sample was assigned a unique research study number by the clinical team disconnecting the sample from personal information of the donor. Women of reproductive age with and without BV symptoms were included.

### Preparation of Transplanted VMB

Vaginal swabs were obtained by clinical research staff during gynecological examinations. The sampling method has been described elsewhere (Medina-Colorado et al., 2017). Briefly, using a standard sterile speculum, a calcium alginate swab (Fisherbrand, Pittsburgh, PA, United States) was passed over the mid-vaginal wall avoiding external sites. The swabs were placed into 2 mL of sterile Ca^++^/Mg^++^ free Dulbecco’s Phosphate Buffered Saline (DPBS; Cellgro, Herndon, VA, United States), transported to the lab at 4°C before being processed. Gram stains were prepared from the swabs for Nugent scoring. Viable VMB aliquots were prepared from the specimen by adding sterile glycerol (10%w/v) as a cryoprotectant to enhance bacterial viability during storage at -80°C as previously reported (Pyles et al., 2014). Additional aliquots were created for molecular studies including DNA extraction via MagNA Pure 96 (Roche; Indianapolis, IN, United States) for characterization by customized qPCR array as previously described (Pyles et al., 2014). Any sample that was molecularly positive for fungi, sexually transmitted pathogens or Y chromosome DNA was excluded from this study (Pyles et al., 2014).

### *sEx vivo* VEC Multilayer Transwell Culture

Immortalized V19 VECs were cultured in either standard monolayer format or in transwell vessels as described previously (Rose et al., 2012). To establish the multilayer cultures, VEC monolayers were released from stock flasks with trypsin, suspended in antibiotic-free Keratinocyte Serum Free Medium (KSFM; Invitrogen, Carlsbad, CA, United States) and (10^5^cells/transwell) added to 96 transwell insert plates (BD Falcon; Franklin Lakes, NJ, United States) or plated in standard culture vessels with or without sterile coverslips for monolayer analyses. For multilayer cultures, the cells were stabilized at 37°C, 5% CO_2_, for 18–24 h after plating in transwells and then the apical medium was removed creating an air-interface. VEC multilayers matured for another 7–10 days with every other day basal chamber replacement of antimicrobial-free KSFM.

### Bacterial Colonization of Multilayer VEC Cultures and Viral Infections

Frozen aliquots of clinical VMB samples, or molecularly qualified monoculture of *Staphylococcus epidermidis* [previously isolated from a clinical VMB (Rose et al., 2012)] were thawed quickly and diluted with DPBS to concentrations of 10^5^–10^6^ total genomes/mL before application of 10 μL to the apical surface of triplicate matured multilayers. VMB communities or the *S. epidermidis* bacteria reached equilibrated levels over the next 24 h (Rose et al., 2012). ZIKV or HSV-2, diluted in KSFM to yield 10^3^–10^4^ PFU/uL for ZIKV or 10^2^ PFU/uL for HSV-2, were then added to the apical surface to model sexual transmission. For these studies, Panamanian (Asianic isolate) and Dakar (African lineage) ZIKV strains (kindly provided by Dr. Alan Barrett, UTMB) were tested in the culture system. HSV-2 strain 186 (Perry et al., 2016) was used for parallel studies in sterile and VMB colonized VEC. Infected or mock-infected cultures were incubated at 37°C, 5% CO_2_ for 24–96 h depending on the study design.

### Immunofluorescent Labeling

V19I VEC were grown on sterile 22 mm^2^ glass coverslips in KSFM to 70% and were then inoculated with 10^4^ PFU of ZIKV or the PBS vehicle and incubated for an additional 48 h and fixed in cold methanol for 4 min. The infected and mock infected cultures were labeled with mouse anti-flavivirus monoclonal antibody (Clone D1-4G2-4-15, EMD Millipore Corporation, Temecula, CA, United States) diluted 1:10 from the purchased stock. Rabbit anti-cytokeratin polyclonal antibody (DAKO, Carpinteria, CA, United States) diluted 1:100 was used as a cell cytoplasmic label. Secondary labeling was accomplished with FITC-labeled donkey anti-mouse and Rhodamine-labeled donkey anti-rabbit antibodies used at 1:250 dilution (KPL Inc., Gaithersburg, MD, United States). Vectashield Hard+Set with DAPI (Vector Laboratories, Burlingame, CA, United States) was used to mount the slips and stain the nucleus of each cell. Both positive and negative conditions were performed in triplicate and imaged on a Zeiss Axiophot outfitted with digital imaging at 32× magnification. The autoexposure feature was used for each condition and slide to obtain an optimal image, impacting the background levels necessary to illustrate a lack of labeling for ZIKV.

### Plaque Titration Assay

Viral titers were determined by plaque titration using African green monkey kidney (Vero) cells as previously described (Herbst-Kralovetz and Pyles, 2006; Shan et al., 2016). Briefly, 60–80% confluent Vero cell monolayers in six well plates (Greiner Bio One; Monroe, NC, United States) were inoculated in triplicate with 10-fold serial dilutions of the ZIKV or HSV-2 stocks. Each plate was rocked for 1 h before a 1% agar/DMEM mixture was added and allowed to solidify at room temperature. The plates were incubated 4–5 days for ZIKV or 48 h for HSV-2 at 37°C, 5% CO_2_ before being stained with a methanol and crystal violet solution (Sigma-Aldrich, St. Louis, MO, United States).

### Molecular Characterization of VMB Bacterial Communities, Viral Genomic Quantification, and Human Beta Defensin Expression Quantification

The contents of each transwell were harvested by lysis with 100 μL of MagNA Pure 96 External lysis buffer (Roche). For ZIKV and human beta defensin samples, RNA was extracted from half of each sample with the MagNA Pure 96 Cellular RNA kit on a MagNA Pure 96 instrument (Roche). RNA was converted immediately to cDNA (iScript; Bio-Rad, Hercules, CA, United States). Quantification of ZIKV genomic material was performed using a primer pair specific for the NS5 gene (Faye et al., 2013). Expression of human beta defensins 1–3 was quantified using primers pair specific for each target (Cho and Blaser, 2012). For VMB and HSV-2 analyses, DNA was extracted from the transwells using the MagNA Pure 96 DNA kit (Roche). HSV-2 genomic titers were established using a qPCR primer pair specific for the gB glycoprotein gene (Namvar et al., 2005; Perry et al., 2016).

Molecular screening of the VMB profile from a sample created by pooling equivalent amounts of DNA from a triplicate set of transwells was completed using a custom qPCR array that quantified 46 of the most common vaginal bacteria as well as total bacterial load (16S) and VEC genomes (human GAPDH) (Medina-Colorado et al., 2017). Organisms of clinical importance in the VMB such as *Lactobacillus* spp., *Gardnerella vaginalis* and it’s sialidase virulence gene, were quantified by individual qPCR assays for every sample (in most cases the initial evaluation and a reproduction for a total of at least six values). Single target qPCR was completed on every well in the study to establish absolute abundance for selected organisms. The bacterial load (log_10_) of selected organisms in uninfected wells was compared to the quantity of each experimental condition for each microbiome. The difference between the experimental and control values was then averaged and statistically compared using the Student’s *t*-test.

### TLR Receptor Agonist

Synthetic TLR agonists tested included lipopolysaccharide (LPS; TLR4) and CpG – ODN 1826 (TL9; Invivogen; San Diego, CA, United States). Recombinant *Salmonella dublin* flagellin (FLAG; TLR5) was produced and purified as previously described (Eaves-Pyles et al., 2001). After VEC fully matured in the transwell cultures, selected concentrations of each agonist in 10 μL of KSFM were added to the apical surface of triplicate wells: CpG (0.1 μg/mL or 0.01 μg/mL), LPS (1 μg/mL or 0.1 μg/mL), FLAG (5 μg/mL or 0.5 μg/mL) as described previously (Rose et al., 2012). ZIKV (1000 PFU) was added 2 h later and incubated at 37°C, 5% CO_2_ each culture was harvested 48 h later and genomic titers were determined.

### Cytokine Quantification

The cell fraction and basal medium were analyzed for cytokines commonly produced by human vaginal epithelia. The ProcartaPlex Human 45-plex kit was used according to the manufacturer’s instructions (Affymetrix eBioscience; Vienna, Austria). Briefly, antibody-bound beads provided in the kit were added to each well of a filter plate and then incubated with 50 μL of each basal chamber sample for at least 1 h at room temperature. After washing with kit-provided buffer, detection antibody was added, and the filter plate was agitated at 500 rpm for 30 min at room temperature. After adding streptavidin-PE, the resulting signals were detected using a Bio-Plex 200 instrument (Bio-Rad, Hercules, CA, United States). Cytokine quantities were extrapolated from a standard curve processed in parallel.

### Data Analyses

For all studies, triplicate transwell cultures were analyzed for each condition and each study was repeated at least once. For microscopy at least five independent fields were evaluated in at least two independent cell cultures. For molecular analyses, quality metrics assessed by qPCR were performed to confirm the state of the VEC multilayer (human GAPDH) and the success of VMB colonization (universal bacterial 16S rDNA), (Pyles et al., 2014). Samples from individual cultures that failed to meet minimal DNA quality cutoffs (at least 10^6^ total GAPDH copies) were excluded from subsequent evaluations. Statistical comparisons for the impact of the VMB on viral replication, the impact of TLR agonists on ZIKV replication and presence of human beta defensins 1–3 were performed using a two-way ANOVA. Changes in cytokine expression were evaluated using Dunnet’s test by comparing the average quantity of selected cytokines in ZIKV-infected, sterile cultures to uninfected, sterile VEC samples. A second analysis was performed using Tukey’s analysis to compare cytokine production by uninfected VMB-colonized VEC to VMB-colonized and then ZIKV infected VEC baselined to ZIKV-infected sterile VEC or sterile uninfected VEC. Outliers were excluded from all data sets that were not in the 99% distribution range. All statistics and graphing analysis were completed using GraphPad Prism v7.0c.

## Results

### ZIKV and HSV-2 Established Infection and Replicated in Cultured Human VEC

**FIGURE 1:**
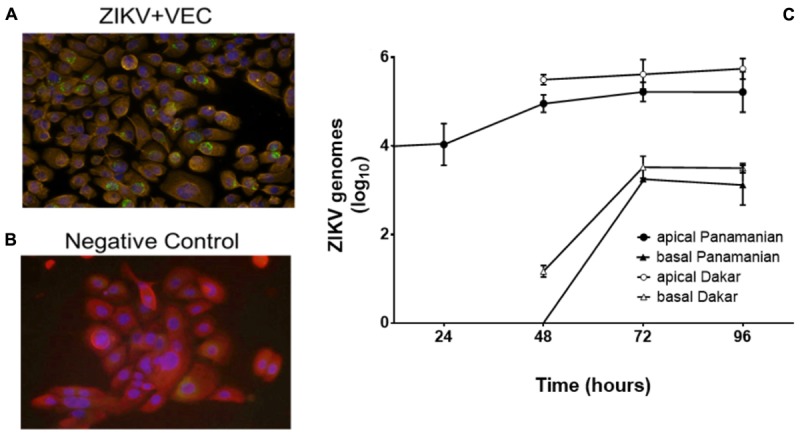
ZIKV replicated in VEC. Immunofluorescent labeling showed the presence of ZIKV (Panamanian) in VEC monolayer cell culture at 48 h post infection (32×). Red = cytokeratin, Blue = cell nucleus (DAPI), Green = ZIKV. **(A)** ZIKV infected cells. **(B)** Negative control. **(C)** Replication kinetics of ZIKV (Panamanian and Dakar) in the mature multilayer VEC culture system (*N* = 6) (average genomes are shown as log_10_ values ±SD). ZIKV in the basal chamber modeled systemic release of the virus from the vaginal mucosa.

In women, sexual transmission of ZIKV likely involves infection of VEC, therefore we first demonstrated that cultured immortalized human V19I VEC supported viral infection and replication. V19I cell monolayers were inoculated with a contemporary Panamanian ZIKV isolate (MOI = 0.1); 48 h later immunofluorescent labeling showed ZIKV in infected (green; Figure 1A) but not uninfected control (Figure 1B) cultures. Further, 72 h after inoculation, infectious ZIKV was recovered from both the culture medium and cell lysates of monolayer cultures at titers 10- to 100-fold higher than the inoculum used (data not shown).

To better model the ZIKV infection of the vaginal mucosa, we performed replication kinetic studies in the VEC multilayer model where cells differentiate and stratify after establishment of an apical air-interface (Pyles et al., 2014). To confirm that ZIKV would infect and replicate in mature multilayer VEC cultures, inoculum containing the two different strains of ZIKV were applied to the apical surface to model sexual transmission and titers were evaluated over 96 h. The 10^4^ ZIKV PFU challenge used was selected to represent viral titers reported in seminal fluid (Mansuy et al., 2016; Turmel et al., 2016). This titer was established considering that each VEC culture represented ∼0.1% of the surface area of the human vagina so we challenged with 0.1% of the reported concentration in a full seminal fluid volume. The Panamanian ZIKV strain represented the Asianic lineage and was compared to the Dakar strain that represented African lineage isolates associated with earlier outbreaks (Simonin et al., 2017). Triplicate VEC multilayers, harvested at 24 h intervals confirmed ZIKV infection and replication with increased genomic titers from 24 through 96 h (Figure 1C). Standard plaque titration in Vero cells confirmed that the detected genomes (qPCR) in both apical and basal fractions were infectious and established a ratio of ∼10 genomes/PFU (Supplementary Figure 1). No statistical difference in replication between the strains was observed. We also evaluated the potential for ZIKV to be released to the basal chamber modeling systemic spread leading to viremia in the host. In the basal chamber that only contained culture medium and no cells, ZIKV was not detected until 48 h with increased titers observed by 96 h. Despite obvious replication, no visible lysis or disruption of the 3D VEC cultures was observed by microscopy or by accumulation of medium from the basal chamber on the apical surface that would indicate a compromised multilayer barrier (Rose et al., 2012).

Evaluation of ZIKV infection in sterile VEC multilayers suggested no obvious inflammation of the VEC was elicited as indicated by a lack of increased levels of 45 quantified cytokines in both the apical and basal chamber samples. In fact, the levels of selected cytokines in medium collected from the basal chamber of ZIKV infected VEC transwells (48 h pi) showed significant decreases in IL-9 (*p* = 0.0002), FGF (*p* = 0.0334), PIGF-1 (*p* = 0.039), and IL-18 (*p* = 0.019) compared to uninfected controls (Supplementary Figures 2A–D). Evaluation of the infected cell fraction showed a significant decrease in IL-8 (*p* = 0.038) (Supplementary Figure 2E). ZIKV infection also had no significant impact on the expression of human beta-defensins 1, 2, or 3 compared to uninfected controls (data not shown). Based on the characterization results, our subsequent evaluations only included the contemporary Panamanian ZIKV at 48 h post infection.

### ZIKV Did Not Impact VMB Composition

**FIGURE 2:**
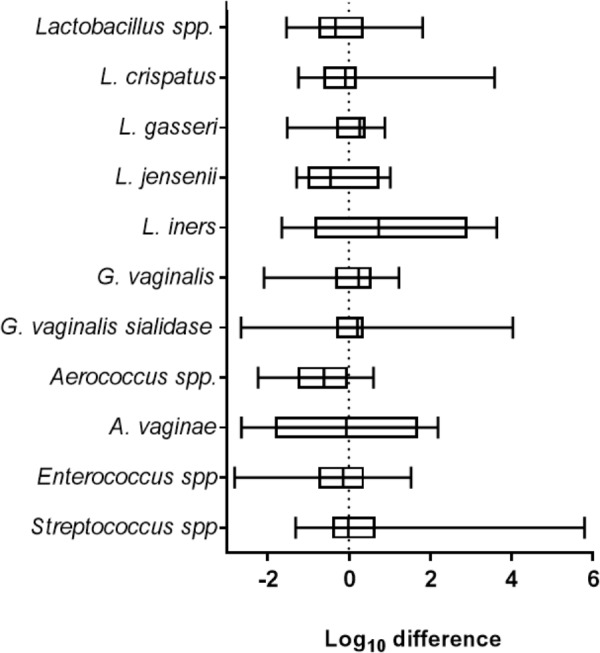
ZIKV infection did not impact the abundance of selected vaginal bacteria. The graph shows the average log_10_ difference (±SD) of the absolute quantity of individual species detected with and without ZIKV infection in all microbiomes tested. There were no significant changes in the quantity of the indicated organism after ZIKV infection. *Lactobacillus* spp., *N* = 28; *L. crispatus*, *N* = 11; *L. gasseri*, *N* = 7; *L. jensenii*, *N* = 10; *L. iners*, *N* = 10; *G. vaginalis*, *N* = 25; *G. vaginalis* sialidase, *N* = 16; *Aerococcus* spp., *N* = 11; *A. vaginae*, *N* = 8; *Enterococcus* spp., *N* = 24; *Streptococcus* spp., *N* = 26. Unpaired Student’s *t*-test analyses were used to compare the VMB colonized control well to the ZIKV infected VMB colonized well.

Clinically, viral STIs have been associated with dysbiosis, inflammation and unhealthy shifts in the VMB (Spear et al., 2008). To determine the impact of ZIKV on the VMB we analyzed changes in selected organism. Evaluation of 35 distinct VMB communities transplanted to mature VEC cultures, was performed to determine the impact of the microbiome on viral replication in VEC multilayers (Supplementary Figure 3). These VMB were selected to study communities dominated by common probiotic and dysbiotic organisms including samples from women with diagnosed BV. We compared the change in quantity of specific target organisms associated with health and inflammation between uninfected and ZIKV infected triplicate cultures by qPCR. The multiple *Lactobacillus* spp. chosen represented the dominant organisms found in microbiomes associated with optimal VMBs (Ravel et al., 2011). *G. vaginalis* and an associated virulence gene, sialidase, have both been highly correlated with unhealthy microbiomes, especially those with symptoms of BV (Martin and Marrazzo, 2016). The log_10_ fold change in absolute abundance of selected targets present in any of the tested microbiomes was calculated and plotted in Figure 2. Remarkably, ZIKV infection did not produce significant changes in absolute abundance of any of the indicated organisms in this model system.

### VMB Communities Impacted ZIKV and HSV-2 Viral Titers

**FIGURE 3:**
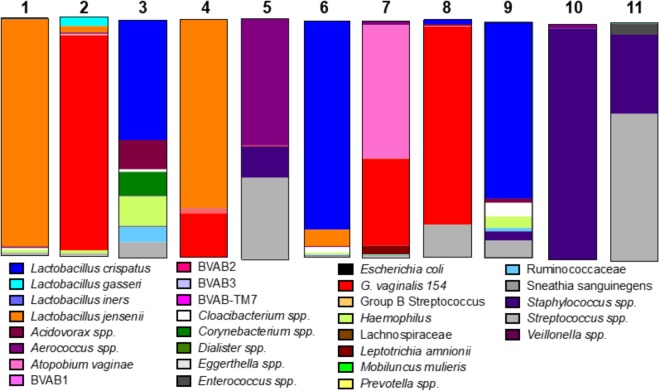
VMB bacterial profiles. The community profiles (average of six replicate cultures) are shown as a relative abundance proportional bar chart compared to the total 16S detected. Each profile was developed using a customized qPCR array. Each target is represented as a different color as shown in the legend.

**FIGURE 4:**
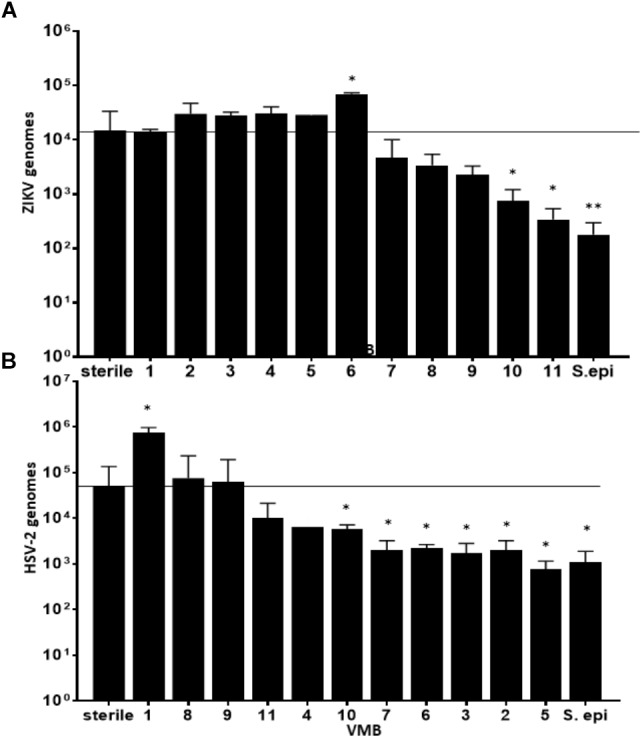
VMBs differently impacted replication of ZIKV and HSV-2. Average ZIKV **(A)** and HSV-2 **(B)** titers (±SD) 48 h post infection in parallel cultures colonized with one of 11 different VMBs (1–11) or a monoculture of *S. epidermidis* (S. epi). Sterile controls were not colonized with a VMB. The horizontal line shows the average ZIKV titer in sterile (no microbiome) VEC. A one-way ANOVA was performed comparing each condition to the sterile control (^∗^*p* ≤ 0.05, ^∗∗^*p* ≤ 0.005).

We considered the previously reported clinical association of dysbiotic communities with acquisition and replication of HIV and HSV-2 (Brotman, 2011) and hypothesized that VMB communities would alter ZIKV replication. However, unlike HIV and HSV-2 there is no clinical data to suggest that certain microbiomes impact ZIKV differently than others. We analyzed the selected 35 VMB communities (Supplementary Figure 3) to determine if any of the microbiomes led to altered replication outcomes for ZIKV or HSV-2 relative to sterile cultures processed in parallel. The results identified 11 VMBs (Figure 3) that impacted either ZIKV or HSV-2 (Figure 4). Interestingly, VMB 6 led to significantly increased ZIKV titers compared to the sterile controls (*p* < 0.05) while this same VMB led to significant reductions in HSV-2 titers (*p* < 0.05). VMBs 2–5 had no impact on ZIKV but were associated with HSV-2 reductions. The communities established by transplant of VMBs 7, 10 and 11 reduced the titers of both viruses (ZIKV 5-fold, 20-fold, and 50-fold, respectively; HSV-2 20-fold, 10-fold, and 8-fold, respectively) although not all of these differences were statistically significant (Figure 4). VMBs 8 and 9 also reduced ZIKV (not significant relative to sterile controls) but had no impact on HSV-2 titers. Finally, VMB 1 led to a 12-fold increase in HSV-2 titer (*p* < 0.05) but had no effect on ZIKV.

To determine the specific effects of VMB composition on increased or decreased viral replication we molecularly profiled VMBs from uninfected wells harvested from cultures created in parallel by our custom qPCR array (Medina-Colorado et al., 2017). Clustering analyses revealed that microbiomes with higher levels of *Staphylococcus epidermidis* (>10^4^ genomic copies) were associated with significantly decreased titers of ZIKV. Figure 4 shows VMBs that associated with decreased ZIKV titers also contained organisms such as *Atopobium vaginae*, *G. vaginalis*, and BVAB2 that are associated clinically with vaginal symptoms and inflammation. Conversely, VMB 6 with increased ZIKV was dominated by *L. crispatus.* This same community decreased HSV-2 consistent with prior clinical associations (Brotman, 2011).

An interesting outcome from these analyses was the association of *S. epidermidis* with reduced titers of ZIKV. In previous studies, we showed that monoculture colonization of VEC multilayers by a *S. epidermidis* isolate we derived from a clinical VMB led to production of specific cytokines and exacerbated responses to TLR agonists (Rose et al., 2012). We therefore tested the impact of this vaginal isolate on ZIKV and HSV-2 infections as a monoculture. Similar to the microbiomes that contained *S. epidermidis*, colonization of VEC multilayers by our vaginal *S. epidermidis* isolate also showed a significant reduction in ZIKV titer (*p* < 0.001) and surprisingly also reduced HSV-2 titer (*p* < 0.05) (Figure 4).

Clustering analyses were less clear for the VMBs that impacted HSV-2. VMB 1, the only microbiome that enhanced HSV-2 titers, was dominated by *L. jensenii*. This was also the only tested VMB in this set that included *Mobiluncus mulieris*. VMBs 2, 3, 5, 6, 7, and 10 all significantly reduced HSV-2 titer compared to the control (*p* < 0.05) but showed few similarities in community profile. These VMBs were only shared commonality in that they contained an abundance of *Lactobacillus* spp.

### Cytokines From Selected VMB/VEC Co-cultures Associated With Increased and Decreased ZIKV Replication

**FIGURE 5:**
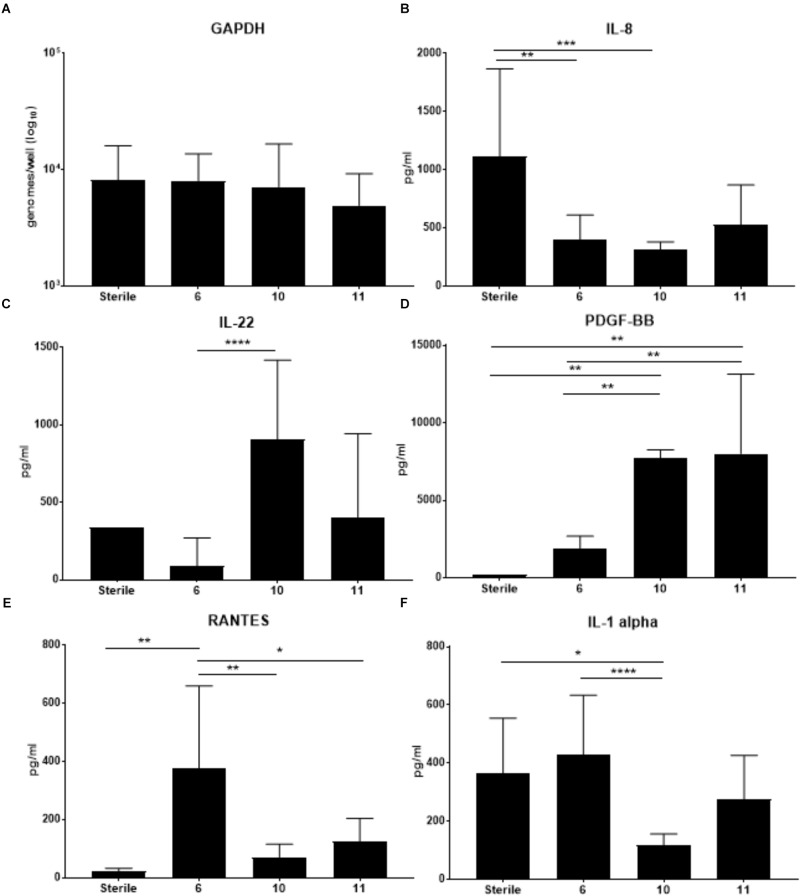
Changes in cytokine production by VEC when colonized with specific VMBs that significantly impacted ZIKV replication. Cytokines were quantitated using a multiplex bead array assay. **(A)** GAPDH levels (established by qPCR) indicated there were no significant impacts on cell counts for the conditions tested. Average levels of selected cytokines (*N* = 6 replicates; ±SD) are illustrated for IL-8 **(B)**; IL-22 **(C)**; PDGF-BB **(D)**; RANTES **(E)** or IL-1alpha **(F)**. Two-way ANOVA analyses between conditions determined significance; ^∗^*p*-value < 0.05, ^∗∗^*p*-value < 0.01, ^∗∗∗^*p*-value < 0.001, ^∗∗∗∗^*p*-value < 0.0001. As a reminder, VMB 6 significantly increased ZIKV replication while VMBs 10 and 11 significantly decreased ZIKV replication.

Previous work from our lab group indicated colonization with *S. epidermidis* led to significantly increased production of several cytokines including IL-1b, IL-8, TNFa, GCSF and IL-1ra in the cell fraction of VEC multilayers (Rose et al., 2012). To determine if VMB communities that contained *S. epidermidis* (VMB 10 and 11) and reduced ZIKV titers also had elevation of these same cytokines, we completed a cytometric bead array (Affymetrix Human-45 plex) on the cell fraction. Additionally, VECs colonized with the VMB that significantly increased ZIKV titers (VMB 6) and sterile (no VMB) VECs, were also evaluated (Figure 5). Analysis of GAPDH was performed to ensure that there was no significance in VEC number between the conditions (Figure 5A).

The cytokine results in Figure 5 indicated that VMB 6 (increased ZIKV) elicited a distinct profile from VMBs 10 and 11 (decreased ZIKV) consistent with a potential role for cytokine production contributing to ZIKV outcomes. Unexpectedly, and in contrast to the previously reported *S. epidermidis* monoculture data, IL-8 was decreased by each of these three VMBs (significantly in VMBs 6 and 10) relative to sterile controls despite the presence of *S. epidermidis* in the VMB 10 and 11 communities (Figure 5B). VMBs that significantly decreased ZIKV (VMB10 and 11) had increased levels of IL-22 and PDGF-BB relative to the VMB 6, and the sterile control (Figures 5C,D). IL-22 was significantly increased in VMB 10 compared to VMB 6. VMB 10 and 11 had significantly higher levels of PDGF-BB compared to the sterile and VMB 6 conditions. This suggests that IL-22 and PDGF-BB both may have a suppressive effect on ZIKV. RANTES was significantly higher in VMB 6 (increased ZIKV) when compared to sterile and VMB 10 and 11 (Figure 5E). IL-1 alpha also was significantly increased in VMB 6 when compared to VMB 10. IL-1 alpha was significantly decreased compared to sterile conditions. While not significant, it was also increased compared to sterile conditions and VMB 11 (Figure 5F). The up or down regulation of cytokines that are consistently associated with VMBs that significantly decrease ZIKV titers indicate pathways that can be further studied to determine their association with resistance to ZIKV infection.

### Activation of TLR Receptors Did Not Significantly Impact ZIKV Titers

Consistent with current and past cytokine data it is clear that the local environment produced by the VMB may have altered susceptibility to infection or replication levels supporting the theory that innate immune responses active at the time of viral exposure may contribute to ZIKV transmission and/or replication. Our previous work with TLR agonists (Rose et al., 2012) applied to the apical surface of VEC multilayers suggested that selected TLR 2/6, TLR 4, TLR 5, and TLR9 agonists might elicit similar innate responses to those engendered by *S. epidermidis*. Treatment of mature sterile VEC multilayers with a low or high dose of the agonists or the DPBS vehicle control for 2 h before apical infection with ZIKV produced a modest reduction in ZIKV titers that was dose dependent when low dose LPS (TLR4) or FLAG (TLR 5) were compared to high dose treatments (Supplementary Figure 4). These results were not significant. Interestingly, CpG (TLR 9) treatment did not show this dose-dependent trend.

### Changes in Human Beta Defensins in Response to a VMB

Human beta defensins play an integral role in preventing infection of the vaginal mucosa by viruses including HSV-2 and HIV (Quiñones-Mateu et al., 2003; Wilson et al., 2013). Previous studies in our VEC model established that matured cultures produced detectable levels of human beta defensins 1, 2, and 3 while human beta defensins 4 and 5 were not detected, therefore they were not evaluated in this study. The results of qRT-PCR for each target did not reveal a correlation between human beta defensin 1, 2, or 3 expression with conditions that were associated with altered ZIKV titer (Supplementary Figure 5). It was noted that the VEC samples colonized by *S. epidermidis* alone had undetectable levels of human beta defensin 1, 2, or 3 gene expression.

## Discussion

At this time, there is little information about ZIKV as a sexually transmitted pathogen in part due to the low number of symptomatic, infected persons (Brooks et al., 2016). Due to the pregnancy and fetal development complications caused by this emerging virus (de Paula Freitas et al., 2016; Sheridan et al., 2017) studies of sexual transmission of ZIKV and careful evaluations of changes in the vaginal mucosa caused by infection are critical. Using our vaginal mucosa culture system, we have confirmed that ZIKV from African and South American genetic lineages infected and replicated in VEC after apical delivery modeling female exposure to infected seminal fluid. Following infection, the virus increased in titer over 48 h before detection in the basal chamber of the culture system predicting the emergence of the pathogen into the blood. Interestingly, ZIKV infection did not appear to be markedly cytotoxic or inflammatory based on a lack of obvious damage to the VEC multilayers and no change in pro-inflammatory cytokine levels including IL-8 and IL1β that are commonly upregulated in inflamed vaginal samples (Thurman et al., 2015; Passmore et al., 2016). ZIKV infection also did not significantly alter expression of human beta-defensins 1, 2, or 3. In contrast, epithelial destruction resulted from HSV-2 infection of the VEC multilayers correlating with clinical symptoms of an active HSV-2 infection (Supplementary Figure 6) (Brotman, 2011; Zhu et al., 2017). Previously published data showed that HSV-2 elicited an inflammatory response in VEC (Herbst and Pyles, 2003).

Clinical studies have established a relationship between the presence of a dysbiotic microbiome and increased viral titers in vaginal secretions in women infected by HSV-2 or HIV (Brotman, 2011; Pyles et al., 2014; Martin and Marrazzo, 2016). Unfortunately, clinical studies do not easily support mechanistic or causation evaluations and are confounded by a variety of behavioral and genetic factors that are difficult to address including sexual preferences, hygiene and diet that impact the VMB community (Marrazzo et al., 2010; Borgdorff et al., 2017; Noyes et al., 2018). In this report, we have completed studies that specifically indicate ZIKV titer is impacted by selected VMB communities. This also was the case for HSV-2 but we involved a distinct set of VMB communities that impacted outcomes for this dsDNA virus. Remarkably, VMB 6 that led to increased ZIKV titers was dominated by *L. crispatus*, an organism associated with vaginal health and resistance to STI acquisition and transmission (DiGiulio, 2017). Other *L. crispatus*-dominated VMBs that were tested did not impact ZIKV titers suggesting that difference in titers among different VMBs could be due to a variety of factors, including but not limited to: differences in minor organisms within the microbiome, differences in the community profile, or bacteriophages (van de Wijgert et al., 2014; Malki et al., 2016).

In contrast, microbiomes associated with significantly decreased ZIKV titers contained levels of *Staphylococcus* spp. greater than 10^4^ genomes. Because *S. epidermidis* was the most commonly found *Staphylococcus* species in these VMBs, we established that a *S. epidermidis* vaginal clinical isolate (Rose et al., 2012) as a monoculture not only decreased the ZIKV titer, but also reduced HSV-2. Quantification of VEC numbers after *S. epidermidis* colonization (Figure 5A) as well as other health measures, such as pooling of medium in the apical chamber indicating loss of tight junctions and/or barrier penetrations, were indistinguishable from parallel sterile VEC cultures (data not shown) suggesting the decrease in ZIKV and HSV-2 titers was not due to VEC damage but rather a yet uncharacterized impact of *S. epidermidis*. It is interesting that *S. epidermidis* and microbiomes containing *Staphylococcus* spp. were able to reduce ZIKV titers; consistent with the predictions from the model, *Staphylococcus* spp. in other mucosal microbiomes has been shown to produce antiviral particles that can prevent infection or replication of infecting viruses (Chen et al., 2016; Zhang and Gallo, 2016). An obvious next step is the investigation of peptides or other metabolites created by *Staphylococcus* spp. in the context of VEC cultures, especially those from *S. epidermidis*.

Clinical data suggest that HSV-2 viral titers are lower in women with *Lactobacillus*-dominated VMBs and, in the case of active infection, these VMBs are associated with lower incidences of recurrent infection when compared to dysbiotic microbiomes and BV (Brotman, 2011; Martin and Marrazzo, 2016). The data from our studies in the vaginal culture model were consistent with these clinical observations showing lower average HSV-2 titers in cultures colonized by VMBs dominated by *Lactobacillus* spp. Previous work and the current studies add validity to the predictive value of this VEC multilayer culture system. It is noted that for these studies, the tested cultures lacked professional immune cells common to the vaginal mucosa that have been successfully added to the model for studies of HIV (Pyles et al., 2014). It is possible that the results for both ZIKV and HSV-2 may be different when macrophage or dendritic cells are included in the VEC multilayers; such questions will be addressed in future studies.

Consistent with the limited perturbation of the VEC environment by ZIKV infection we observed that the infection did not significantly alter the quantity of any tested organisms common to the VMB. Viral infections of VEC, especially HSV-2 and HIV, typically alter cellular functions and modulate gene expression leading to predictable changes in a variety of metabolites, cellular receptors and transporters that, in turn, impact the VMB (Ghartey et al., 2015; Srinivasan et al., 2015). Conversely, the presence of specific VMB communities did lead to altered ZIKV and HSV-2 titers suggesting that the environment, present at the time of exposure, may alter antiviral conditions. Analysis of the cytokines produced by VEC impacted by VMB colonization indicated that IL-22 appeared inversely related to ZIKV titers; IL-22 was increased in both of the VMBs that decreased ZIKV replication (VMB 10 and 11) and IL-22 was decreased in the VMB that increased ZIKV titers (VMB 6). IL-22 has been associated with increased levels of antimicrobial peptides produced by epithelial cells and protection and regeneration of the epithelial barrier (Valeri and Raffatellu, 2016). PDGF-BB also was significantly increased in both of the VMBs that decreased ZIKV titers. While the impact and role of PDGF-BB in vaginal epithelial tissue is relatively unknown, it has been documented as important for establishment and maintenance of growth and integrity of vaginal tissue (Zhang et al., 2017). It has been reported that PDGF-BB serum levels were reduced in ZIKV-infected persons in endemic areas of South America (Barros et al., 2018) when compared to levels of uninfected persons but this may not be relevant to a local (vaginal) site of infection or vaginal responses. Future studies of selective inhibition or supplementation of IL-22 and PDGF-BB in our culture system will help to address their actual contribution to inhibition of ZIKV infection of the vaginal mucosa.

Similarly, we investigated whether selected VMB communities could enhance ZIKV infection or replication, identifying a community (VMB 6) dominated by *L. crispatus* that led to significantly higher titers of ZIKV (Figure 4). In VMB 6 colonized cultures produced significantly higher levels of RANTES and IL-1 alpha were observed compared to VEC cultures colonized by VMB 10 or 11 (that were characterized by decreased ZIKV replication). IL-1 alpha is considered a pro-inflammatory cytokine that is associated with vaginal infection and inflammation (Alcaide et al., 2017). While in many cases these pro-inflammatory cytokines serve as a protective mechanism against infection there are examples of exploitation of increased inflammation to enhance cell to cell spread of virus or enhanced replication including HIV-1 vaginal infection (Naranbhai et al., 2012). Unexpectedly, IL-8, a common marker of vaginal inflammation (Thurman et al., 2015), was decreased by all three VMBs that impacted ZIKV titers. This finding highlights the complex nature of the relationship between the bacterial community and the vaginal mucosa and suggests that not all pro-inflammatory cytokines play a direct role in regulating ZIKV titers.

In an attempt to create distinct inflammatory states in VEC prior to ZIKV infection we utilized sterile cultures treated with selected TLR agonists as previously reported (Rose et al., 2012) to test the theory that VMB activation of TLRs may impact ZIKV outcomes. As executed, no statistical differences in ZIKV titer relative to untreated controls were revealed, however, a trend of dose-dependent, reduced viral genomic content following treatment with FLAG motifs (TLR5) or LPS (TLR4) was observed. It is important to note that we consistently tested a single time point (48 h) following ZIKV infection; earlier time points after infection likely would reveal a different profile of responses and outcomes. As noted, these studies were completed without the addition of immune cells creating an additional limitation for interpretation of these initial studies. We chose to exclude these cells in order to determine the most fundamental impacts of ZIKV on vaginal epithelia and associated VMB. Finally, our analysis of the VMB was qPCR-based analyzing the impact of only those bacterial targets included in the custom array thereby missing potentially important contributors to the viral outcomes. In general, the array was able to cover >90% of each tested microbiome’s bacterial content suggesting any missed organism was less than 10% of the total population in the community. Nevertheless, these organisms may play a critical role in the observed phenotypes.

In summary, use of the VEC culture system predicts that the VMB present at time of exposure to ZIKV and HSV-2 can significantly influence viral outcomes. Creation of the multilayers and subsequent colonization by transplanted VMBs or organisms in a 5% CO_2_ supplemented atmospheric air was utilized to model the vaginal lumen as previously described (Medina-Colorado et al., 2017). Further, investigation of these sexually transmitted viruses in this culture model will allow for better understanding of the mechanism and direct interactions involved and should elucidate key pathways and molecules that could serve as future preventatives or therapeutics. This system also should allow testing of both safety and efficacy of vaginally applied medications on the VEC and VMB for development of methods to protect women and fetuses from malformations and sequelae caused by ZIKV and HSV-2.

## Author Contributions

MA-B, MW, CM, and AM planned and carried out the experiments. MA-B and KV obtained informed consent and collected vaginal swabs. MA-B and RP analyzed the data. MA-B wrote the manuscript with support from KV, NB, and RP. NB and RP supervised the project.

## Conflict of Interest Statement

KV and RP are paid consultants to ABL, Inc The remaining authors declare that the research was conducted in the absence of any commercial or financial relationships that could be construed as a potential conflict of interest.
