# Supplementary figures and images for: Cultivated Human Vaginal Microbiome Communities Impact Zika and Herpes Simplex Virus Replication in ex vivo Vaginal Mucosal Cultures
Source: Front Microbiol. 2019 Jan 14;9:3340. doi: 10.3389/fmicb.2018.03340 (PMC6340164; doi:10.3389/fmicb.2018.03340)

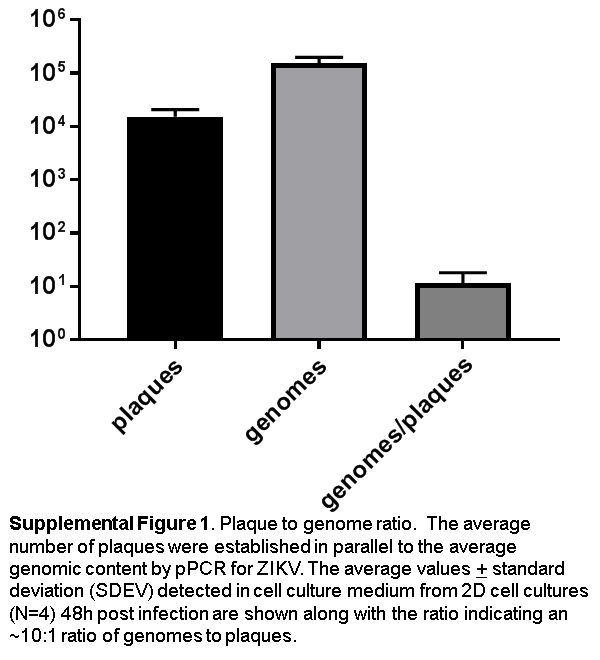

Supplement: Supplementary file 1 [file Image_1.TIF]

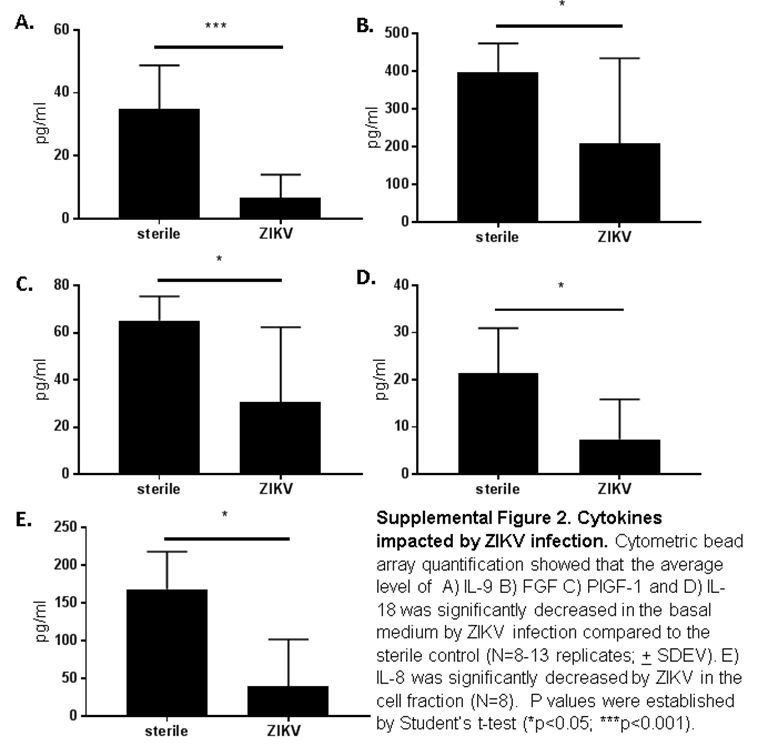

Supplement: Supplementary file 2 [file Image_2.TIF]

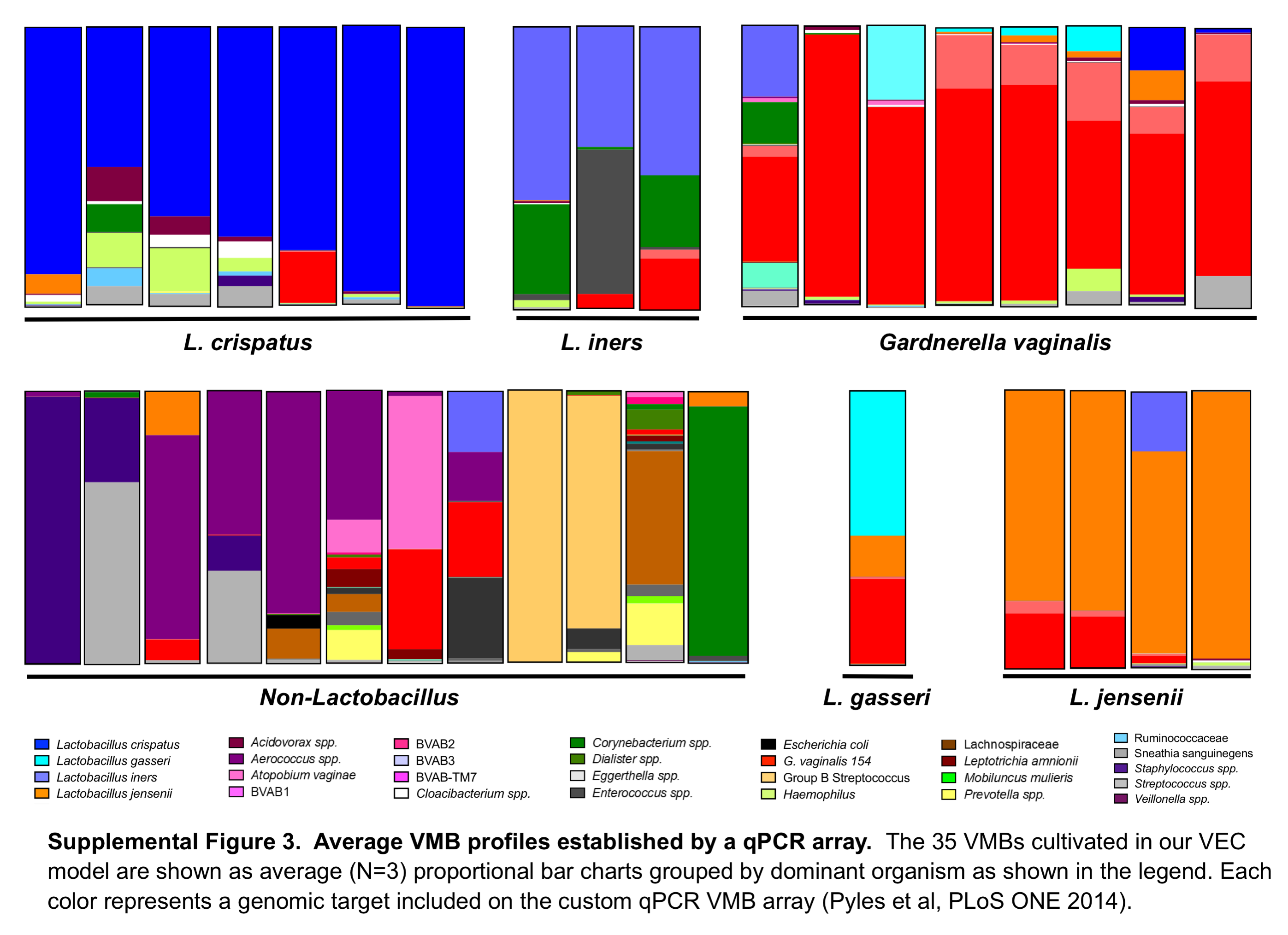

Supplement: Supplementary file 3 [file Image_3.TIF]

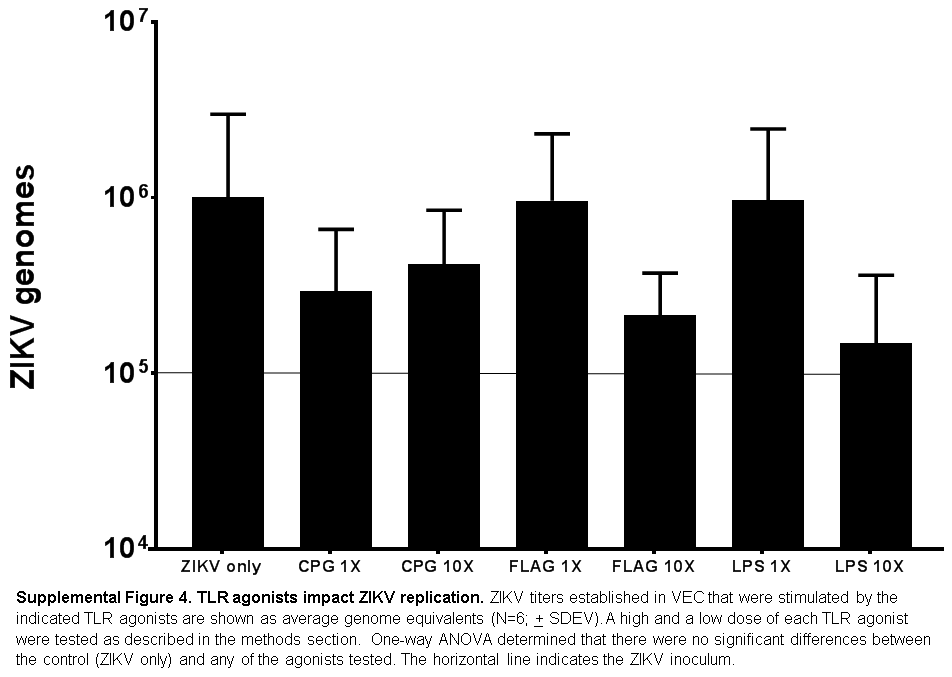

Supplement: Supplementary file 4 [file Image_4.TIF]

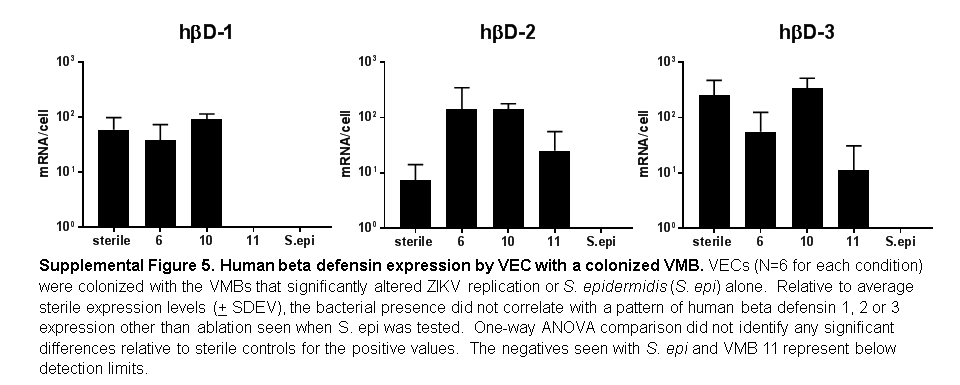

Supplement: Supplementary file 5 [file Image_5.TIF]

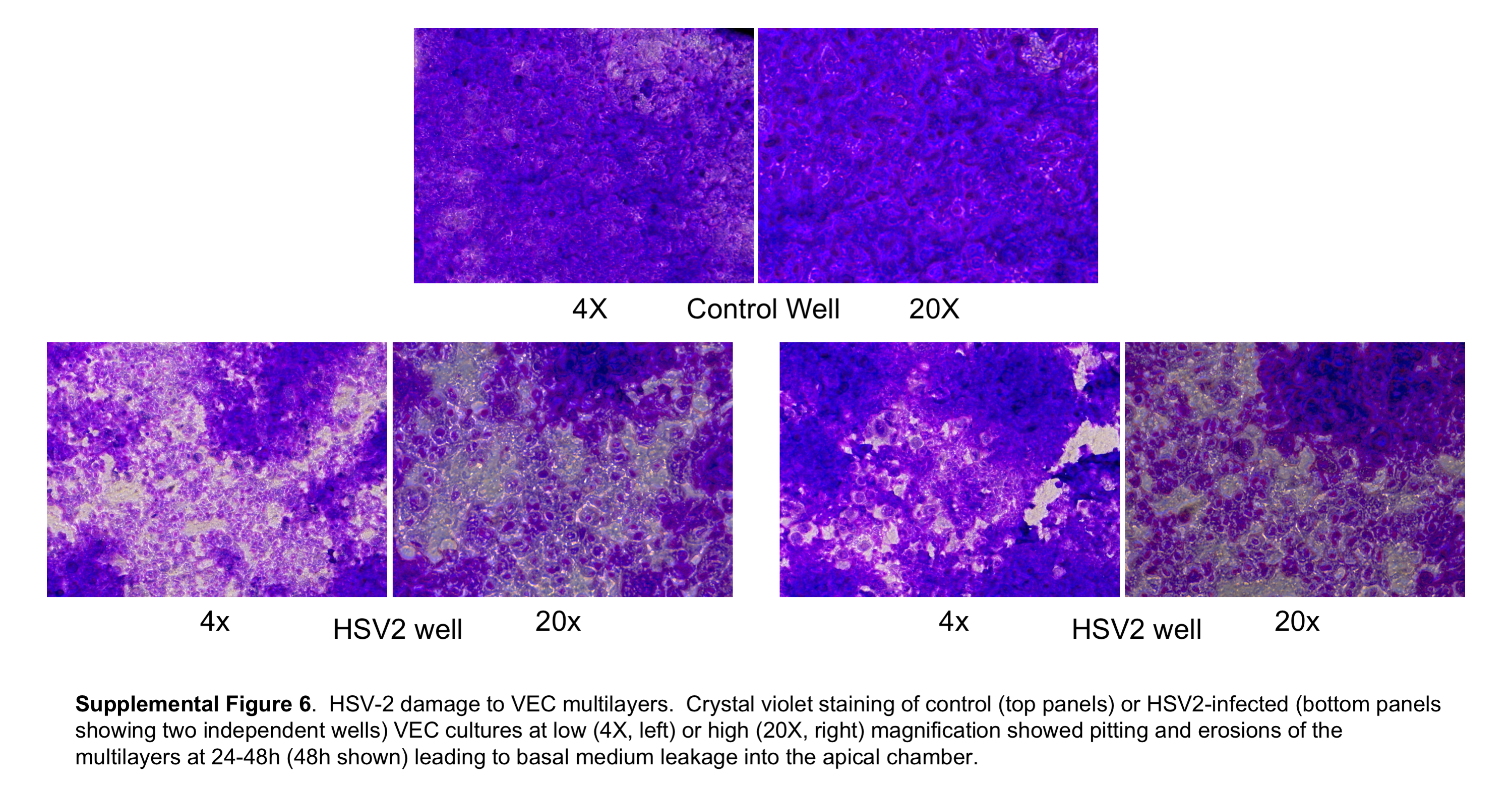

Supplement: Supplementary file 6 [file Image_6.TIF]
